# The impact of task relevance and degree of distraction on stimulus processing

**DOI:** 10.1186/1471-2202-14-107

**Published:** 2013-10-01

**Authors:** Stefanie C Biehl, Ann-Christine Ehlis, Laura D Müller, Andrea Niklaus, Paul Pauli, Martin J Herrmann

**Affiliations:** 1 Department of Psychiatry, Psychosomatics and Psychotherapy, University of Würzburg, Füchsleinstraβe 15, 97080 Würzburg, Germany; 2 School of Psychology, University of Aberdeen, William Guild Building, Aberdeen AB24 3FX, UK; 3 Department of Psychiatry and Psychotherapy, University of Tuebingen, Osianderstraβe 24, 72076 Tuebingen, Germany; 4 Department of Psychology I, University of Würzburg, Marcusstraβe 9-11, 97070 Würzburg, Germany; 5 Department of Human Genetics, University of Würzburg, Am Hubland, 97074 Würzburg, Germany

**Keywords:** Selective attention, Working memory, Cognitive control, P100, N170, ADHD

## Abstract

**Background:**

The impact of task relevance on event-related potential amplitudes of early visual processing was previously demonstrated. Study designs, however, differ greatly, not allowing simultaneous investigation of how both degree of distraction and task relevance influence processing variations. In our study, we combined different features of previous tasks. We used a modified 1-back task in which task relevant and task irrelevant stimuli were alternately presented. The task irrelevant stimuli could be from the same or from a different category as the task relevant stimuli, thereby producing high and low distracting task irrelevant stimuli. In addition, the paradigm comprised a passive viewing condition. Thus, our paradigm enabled us to compare the processing of task relevant stimuli, task irrelevant stimuli with differing degrees of distraction, and passively viewed stimuli. EEG data from twenty participants was collected and mean P100 and N170 amplitudes were analyzed. Furthermore, a potential connection of stimulus processing and symptoms of attention deficit hyperactivity disorder (ADHD) was investigated.

**Results:**

Our results show a modulation of peak N170 amplitudes by task relevance. N170 amplitudes to task relevant stimuli were significantly higher than to high distracting task irrelevant or passively viewed stimuli. In addition, amplitudes to low distracting task irrelevant stimuli were significantly higher than to high distracting stimuli. N170 amplitudes to passively viewed stimuli were not significantly different from either kind of task irrelevant stimuli. Participants with more symptoms of hyperactivity and impulsivity showed decreased N170 amplitudes across all task conditions. On a behavioral level, lower N170 enhancement efficiency was significantly correlated with false alarm responses.

**Conclusions:**

Our results point to a processing enhancement of task relevant stimuli. Unlike P100 amplitudes, N170 amplitudes were strongly influenced by enhancement and enhancement efficiency seemed to have direct behavioral consequences. These findings have potential implications for models of clinical disorders affecting selective attention, especially ADHD.

## Background

The ability to suppress the processing of irrelevant and thereby distracting information is of paramount importance in daily life and the search for its neural correlates has drawn much attention. An influential study using a delayed recognition task found a suppression of visual processing when a stimulus was not task relevant and an enhancement when it was [1]: Functional magnetic resonance imaging (fMRI) showed changing activation in fusiform and parahippocampal regions of interest (ROIs) depending on task relevance. The same study also recorded EEG and investigated P100 and N170 peak amplitudes. The N170 is an event-related potential (ERP) of early visual processing that is particularly sensitive to facial stimuli [2]. An influential study by Vogel and Luck [3] furthermore showed the posterior N1 – which peaked around 160 ms – to be sensitive to visual discrimination demands of a task. The P100 was previously shown to be influenced by (spatial) selective attention processes [4-7]. Gazzaley and colleagues [1] found increased peak amplitudes of the N170 when task relevant face stimuli compared to task irrelevant face stimuli were presented. In contrast, P100 peak amplitudes were not significantly altered by task relevance.

The finding of enhanced N170 amplitudes for task relevant stimuli was generalized to a variation of the n-back task. The classic n-back task consists of presenting participants with a sequential string of letters. Participants are then instructed to press a response key whenever a letter is identical to the letter shown ‘n’ (i.e. one, two, or more) trials earlier see for example [8]. Schreppel and colleagues [9] altered this n-back task by presenting task relevant stimuli interspersed with low distracting task irrelevant stimuli. They found enhanced N170 amplitudes for task relevant compared to task irrelevant and passively viewed stimuli. Task irrelevant and passively viewed stimuli were not significantly different from each other. In addition, task relevance seemed to influence P100 amplitudes as well, with task relevant stimuli leading to higher amplitudes than passively viewed stimuli. An effect of task relevance on P100 amplitude was also found in another study, in which it seemed to be connected to working memory performance [10].

Another EEG study examined the N170 in a delayed recognition paradigm with distractors placed in between a to-be-remembered stimulus and the to-be-recognized item [11]. This study found a larger reduction of N170 amplitudes when a distractor was from the same category as the to-be-remembered stimulus (high distracting) compared to when it was from a different category (low distracting), whereas P100 amplitudes did not seem to differ with the degree of distraction. These findings indicate that the more similar a distractor is to a target stimulus the more its processing is suppressed. However, since the study did not include a passive viewing “baseline” condition, no definite conclusion about suppression and/or enhancement processes can be made.

Modulation of visual processing was shown to be affected by frontal lobe functioning some years ago [12,13], and the concept of cognitive control [14] might provide a theoretical framework to explain the variations described above. Egner and Hirsch [15] point to a model originally stemming from research on error processing [16]. This model suggests a system that regulates attentional resources by ‘conflict-monitoring’ on the one hand (mediated by the anterior cingulate cortex (ACC)) and ‘cognitive control’ on the other hand (mediated by the dorsolateral prefrontal cortex (DLPFC)), and has later been confirmed by findings from neuroimaging research [17]. Several other studies with non-simultaneous stimulus presentation also report an involvement of frontal areas in distractor suppression [18-21].

To summarize, it appears that relevance-induced peak amplitude differences are fairly consistent across paradigms. Several studies show enhanced or suppressed P100 and/or N170 amplitudes depending on the relevance of a processed stimulus or its degree of distraction. These studies, however, differ greatly in employed paradigm, with some studies relying on simultaneous presentation of both task relevant and distracting stimuli [10] or a Stroop-like design [15,22] while others present task relevant and task irrelevant stimuli in sequential order [1,9,11,23]. The thereby created conditions are also dissimilar across studies: While all studies contain task relevant stimuli, the task irrelevant stimuli differ in their degree of distraction [11] depending on whether the stimuli are from the same or a different category as the task relevant stimuli [1,9,10]. In addition, only some studies include a passive viewing baseline condition [1,9,10,23] that seems to lead to somewhat intermediate activation. To our knowledge, no study so far included both high and low distracting task irrelevant stimuli as well as a passive viewing baseline condition.

**Figure 1:**
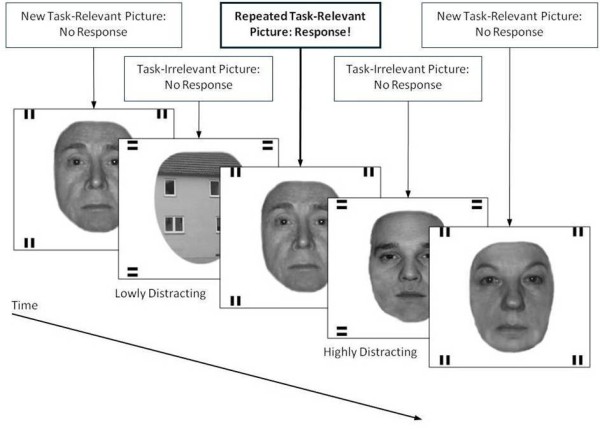
**The experimental paradigm (an example of the “faces relevant” condition): Relevant stimuli are marked by vertical bars; irrelevant stimuli are marked by horizontal bars.** Stimuli were presented for 1000 ms. A grey fixation cross was shown for 1,750 ms to 2,750 ms in between stimuli. Participants were supposed to indicate when a task relevant picture was repeated 1-back while ignoring the interspersed task irrelevant distractors.

In order to compare passively viewed stimuli to task relevant and to high and low distracting task irrelevant stimuli, we decided to combine several aspects of the above-mentioned tasks: We used a modified 1-back paradigm similar to the one used by Schreppel and colleagues [9] described above to investigate both P100 and N170 amplitudes. In our experimental paradigm, task relevant and task irrelevant stimuli were alternately presented in sequential order. We used stimuli from two different categories (faces and houses). In each block, 50% of the task irrelevant stimuli were from the same category as the task relevant stimuli (and thereby high distracting), and 50% were from another category (and thereby low distracting). Participants had to indicate if a task relevant stimulus was repeated 1-back while ignoring the interspersed task irrelevant distractors (see Figure 1). In addition, we included a passive viewing baseline condition that did not require any behavioral response. The structure of our paradigm provided by the underlying n-back task allows for the examination of continuous attentional processes, which differentiates this paradigm from previous investigations using delayed recognition paradigms. The continuous nature of our task might be conducive to a more stable attentional set than the delayed recognition paradigms where attention necessarily fluctuates between trials. In addition, the behavioral data obtained in our paradigm can easily be related to both impulsivity (i.e. false alarms) and inattention (i.e. missed targets) making this paradigm especially suitable for the assessment of participants with problems in the area of attention regulation.

We furthermore assessed subclinical symptoms of ADHD, a disorder that was previously connected to problems with top-down distractor suppression and cognitive control [24,25] as well as deficient DLPFC functioning [26-28]. The goal was to explore whether distractor processing might vary systematically with the amount of reported ADHD symptoms.

Based on previous results, we expected task relevant stimuli to lead to enhanced amplitudes and high distracting task irrelevant stimuli to lead to reduced amplitudes relative to the passive viewing baseline condition [1,9]. In line with previous studies, we further expected low distracting task irrelevant stimuli to lead to significantly less suppression and thereby higher amplitudes than high distracting task irrelevant stimuli [11]. Given reports of easy distractibility in patients with ADHD, we also calculated exploratory correlations between mean amplitudes across conditions, behavioral parameters, and participants’ scores on three CAARS self-report scales incorporating typical symptoms of adult ADHD.

## Results

### Behavioral results

All participants detected at least 50 percent of the target trials. The average rate of detected targets was 88.8 percent (*SD* = 9.9), the average reaction time was 741 ms (*SD* = 123; see Table 1 for further sample characteristics). The rate of missed targets and false alarms as well as the average reaction time were not significantly different for face versus house stimuli (all *p* > .1).

**Table 1:** Sample characteristics: mean M (standard deviation SD)

	***M (SD)***
*CAARS (T-scores)*	
Inattention/Memory Problems	42.2 (7.9)
Hyperactivity/Restlessness	44.2 (9.7)
Impulsivity/Emotional Lability	43.8 (8.8)
*PANAS*	
Positive affect	19.7 (6.3)
Negative affect	2.7 (4.4)
*BDI2 (sum score)*	6.2 (6.7)
*Cigarettes per day*	1.0 (4.5)
*Behavioral data*	
% Missed targets	11.3 (9.9)
False alarms	5.4 (3.5)
Reaction time (in milliseconds)	741 (123)
*Usable epochs*	
Task relevant stimuli*	120.3 (9.2)
Task irrelevant, high distracting**	77.6 (4.7)
Task irrelevant, low distracting**	77.1 (6.4)
Passive viewing**	78.4 (3.4)

*Note.* *out of 128 epochs; **out of 80 epochs.

### EEG results

**Figure 2:**
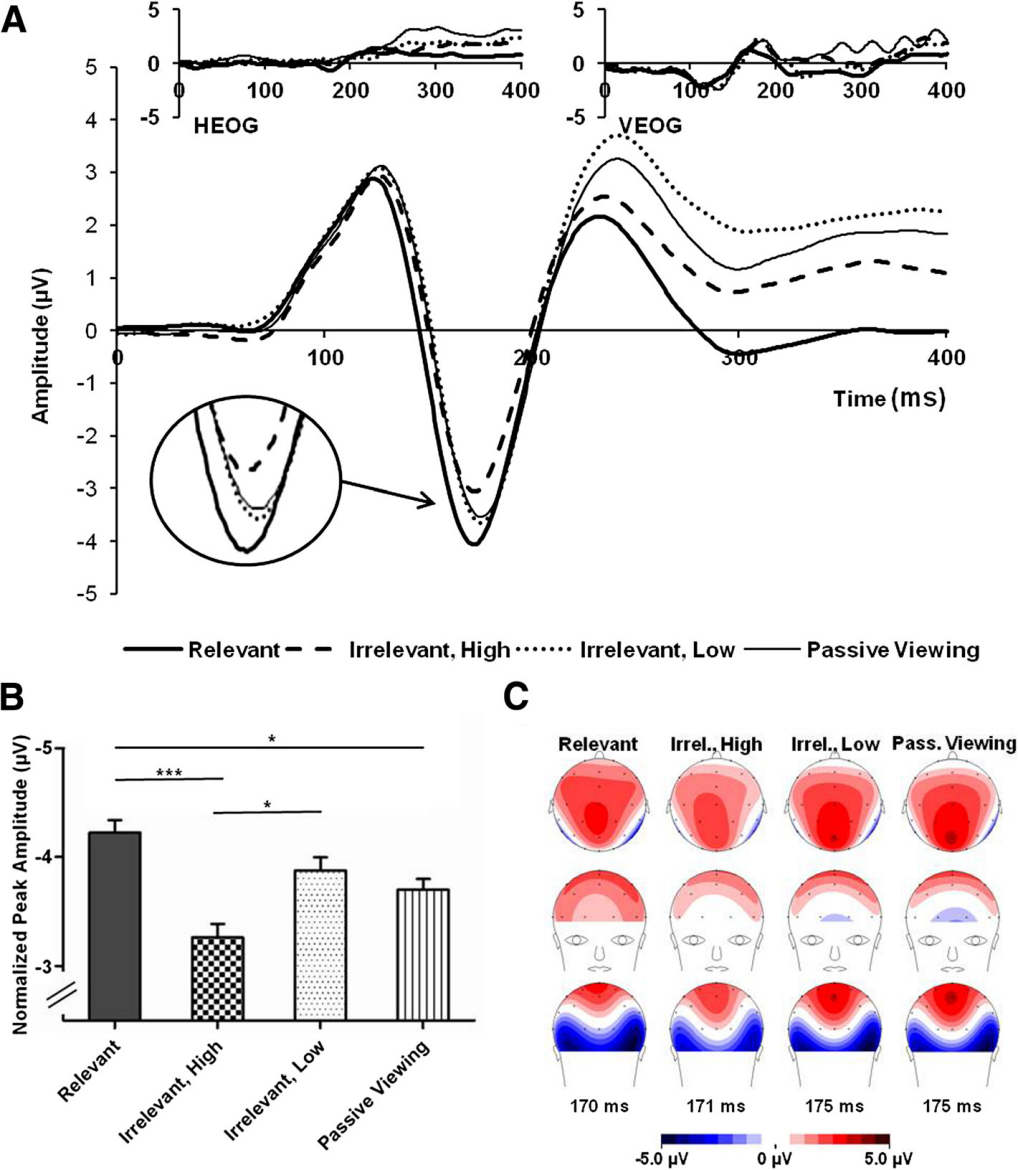
**N170 EEG results. A**. N170 grand average time course over electrodes P7/P8 and PO9/PO10 for the different conditions. Horizontal (HEOG) and vertical (VEOG) electrooculogram activity is displayed in the upper part of the figure showing that eye movements were insignificant and did not differ across conditions. **B**. Mean N170 peak amplitudes for the different conditions. Data were normalized using the *normalization method* described in Franz and Loftus [60] to remove irrelevant between-subjects differences. Error bars denote standard error of the mean for the normalized scores (*SEM*^norm^). **C**. Grand average topographies at N170 peak for the different conditions. Scaling is the same across all conditions. Note.*p < .05, ***p < .001.

We analyzed the P100 and the N170 using repeated measures analyses of variance (ANOVAs). In addition, a post-hoc analysis of the P200 was conducted, as an examination of grand average waveforms pointed to strong task-related modulations of the P200 (see Figure 2A), which seemed worth investigating. The ANOVAs always comprised the within-subject factors hemisphere (left, right), stimulus category (face, house), and task relevance (relevant, irrelevant – high distracting, irrelevant – low distracting, passively viewed), with the additional factor channel group (P7/P8, PO9/PO10) for the analyses of the N170 and P200. The P100 was defined as the most positive peak in the channels O1/O2 in the time window from 70 ms to 140 ms, the N170 was defined as the most negative peak in the channels P7/P8 and PO9/PO10 in the time window from 140 ms to 210 ms, and the P200 defined as the mean amplitude in the time window from 210 ms to 270 ms in the channels P7/P8 and PO9/PO10 (see Figure 2A.). Correlation coefficients were calculated for the mean ERPs across the four task conditions and the three CAARS subscales, between difference amplitudes of task relevant minus high distracting task irrelevant stimuli and behavioral measures, and between behavioral measures and the CAARS subscales.

### P100

The repeated measures ANOVA with the factors hemisphere, stimulus category, and task relevance yielded a significant main effect of stimulus category (*F*_(1,19)_ = 7.2, *p* = .02), with significantly higher amplitudes for face compared to house stimuli. The ANOVA showed no further significant main effects or interactions.

### N170

The repeated measures ANOVA with the factors hemisphere, channel group, stimulus category, and task relevance yielded a significant main effect of task relevance (*F*_(3,57)_ = 9.1, *p* < .001). Across the channel groups, task relevant stimuli led to significantly higher peak amplitudes than high distracting task irrelevant stimuli (*p* < .001, *d* = 1.31) and to significantly higher peak amplitudes than passively viewed stimuli (*p* = .03, *d* = 0.65; see Figure 2 for mean amplitudes in the different conditions and for time courses and grand average topographies). Low distracting task irrelevant stimuli yielded significantly higher amplitudes than high distracting task irrelevant stimuli (*p* = .03, *d* = 0.63), but were not significantly different from task relevant stimuli (*p* = .28) and from passively viewed stimuli (*p* = .36). Amplitudes for high distracting task irrelevant stimuli and for passively viewed stimuli were also not significantly different (*p* = .12).

In addition, there was a significant main effect of stimulus category (*F*_(1,19)_ = 213.5, *p* < .001) with face stimuli yielding significantly higher amplitudes than house stimuli across all channels and conditions. A significant interaction of hemisphere and channel group (*F*_(1,19)_ = 5.8, *p* = .03) yielded no significant post-hoc differences. There was no significant interaction of channel group and task relevance (*F*_(3,57)_ = 2.5, *p* = .07) or of stimulus category and task relevance (*F*_(3,57)_ = 2.4, *p* = .08). An analysis of mean amplitudes instead of peak amplitudes also showed a significant main effect of task relevance (*F*_(3,57)_ = 8.1, *p* = .001) with a similar albeit weaker pattern of post-hoc results.

### P200 post-hoc analysis

A repeated measures ANOVA with the factors hemisphere, channel group, stimulus category, and task relevance yielded significant main effects of hemisphere (*F*_(1,19)_ = 5.2, *p* = .03), channel group (*F*_(1,19)_ = 13.4, *p* = .002), stimulus category (*F*_(1,19)_ = 13.4, *p* = .002), and task relevance (*F*_(3,57)_ = 30.4, *p* < .001): Mean amplitudes were significantly higher in the right hemisphere than in the left hemisphere, in the P channel group than in the PO channel group, and for houses than for faces. There were also significant interactions of hemisphere and channel group (*F*_(1,19)_ = 6.0, *p* = .02) and of stimulus category and task relevance (*F*_(3,57)_ = 6.3, *p* = .004): Post-hoc paired t-tests showed significant hemisphere differences for the P channel group with significantly higher amplitudes for P8 than for P7 (*t*_(19)_ = 2.5, *p* = .02), which were not present in the PO channel group. Furthermore, post-hoc paired t-tests showed significantly lower mean amplitudes for relevant faces than for high distracting faces (*t*_(19)_ = 3.5, *p* = .002), for low distracting faces (*t*_(19)_ = 3.4, *p* = .003), and for passively viewed faces (*t*_(19)_ = 3.4, *p* = .003) with no further differences between stimuli. In contrast, the house stimuli were different from each other for most of the analyzed conditions (all *p* < .008, except for relevant and high distracting stimuli: *p* = .08), with amplitudes for relevant stimuli being the lowest, followed by high distracting stimuli, passively viewed stimuli, and low distracting stimuli. There were no further significant main effects or interactions.

### Correlations

The N170 difference amplitude of task relevant minus high distracting task irrelevant stimuli was significantly correlated with the number of false alarms (*r*_(18)_ = .52, *p* = .02): The smaller the difference between these amplitudes (i.e. the less processing enhancement for task relevant stimuli compared to high distracting task irrelevant stimuli), the more false alarms were committed.

The CAARS ‘Hyperactivity/Restlessness’ and ‘Impulsivity/Emotional Lability’ subscales correlated significantly with pooled N170 amplitudes (Hyperactivity/Restlessness’: *r*_(18)_ = .48, *p* = .03; ‘Impulsivity/Emotional Lability’: *r*_(18)_ = .56, *p* = .01): The higher participants’ symptoms on these subscales, the less negative their N170 amplitudes across all task conditions.

In addition, the number of missed target trials correlated significantly with participants’ scores on the CAARS ‘Inattention/Memory Problems’ and ‘Hyperactivity/Restlessness’ subscales: The higher the number of missed target trials, the higher participants scored on those subscales (‘Inattention/Memory Problems’: *r*_(18)_ = .53, *p* = .02; ‘Hyperactivity/Restlessness’: *r*_(18)_ = .55, *p* = .01). In contrast, the number of false alarms correlated significantly with participants’ scores on the CAARS ‘Impulsivity/Emotional Lability’ subscale: The higher the number of false alarms, the higher participants scored on this subscale (‘Impulsivity/Emotional Lability’: *r*_(18)_ = .46, *p* = .04).

## Discussion

We found a modulation of N170 amplitude by the task relevance of the presented stimuli. Since our paradigm enabled us to vary the degree of distraction caused by the task irrelevant stimuli, we found an interesting dissociation that clearly extends previous findings: Amplitudes were significantly higher for task relevant than for high distracting task irrelevant and for passively viewed stimuli, while there was no difference between task relevant and low distracting stimuli. In addition, the amplitudes for low distracting stimuli were significantly higher than for high distracting stimuli. At the same time, the amplitudes for all distracting stimuli were not different from the amplitudes for passively viewed stimuli. Amplitudes of the P100, however, were not significantly influenced by stimulus relevance.

Our pattern of results for the N170 points to a processing enhancement for task relevant stimuli compared to a passive viewing baseline. This enhancement seems to be reduced or possibly even absent for low distracting task irrelevant stimuli, which did not differ from passively viewed stimuli. In addition, amplitudes to high distracting stimuli were significantly lower than to low distracting stimuli. Visual inspection of the grand average waveforms indicates the possibility that the processing of high distracting stimuli might even have been suppressed below the passive viewing baseline. However, as this potential amplitude reduction did not pass the significance threshold after correcting for multiple testing, the notion of distractor suppression cannot be supported by our results. In contrast, the results could also be interpreted as representing different levels of processing enhancement that was influenced by how distracting a task irrelevant stimulus was.

The enhancement of processing for task relevant stimuli compared to high distracting task irrelevant stimuli seems to be directly related to task performance: Small N170 difference amplitudes for these stimuli – indicating less effective enhancement –correlated positively with the number of false alarms. Participants with less effective processing enhancement were possibly more distracted by the task irrelevant stimuli, which interfered with successful working memory maintenance of the task relevant 1-back picture and led to more false alarms. In fact, a paired t-test also yielded significantly higher mean amplitudes to those task irrelevant stimuli followed by false alarms than to task irrelevant stimuli not followed by false alarms. Although only few trials entered this analysis because the number of false alarms across participants was rather low, this difference could support two different explanations: Either the processing of distracting stimuli was usually suppressed, and when it was not participants often were distracted enough to make a false alarm response to the following task relevant stimulus. Or the processing of these task irrelevant stimuli was “accidentally” enhanced, which made these stimuli more distracting and then caused false alarms later on.

Processing of the high distracting task irrelevant stimuli was possibly influenced by the DLPFC ‘cognitive control’ aspect of the neural network proposed by Cohen and colleagues [16]. This ‘cognitive control’ aspect might have been activated as soon as the ACC ‘conflict-monitoring’ part of the network signaled high ‘conflict’ caused by the high distracting stimuli. Another possible explanation for the lower amplitudes to high distracting stimuli might be that each high distracting stimulus has to compete for processing resources with the task relevant stimulus that is being maintained in working memory. Several studies show that simultaneous presentation of stimuli that activate the same neural populations led to decreased ERPs for the stimuli that were not directly task relevant [29,30]. In addition, the ERPs to task relevant target stimuli were found to be reduced when working memory load was increased from maintaining one face to maintaining two or more faces [31]. Since task relevant and high distracting task irrelevant stimuli in our study were from the same category, they likely activated the same neural networks which might have caused the high distracting stimuli to evoke lower event-related potentials. However, stimuli in our study were subsequently (and not simultaneously) presented and working memory load consisted of only one task relevant stimulus at a time. Although this rather low load is possibly not substantial enough to invoke the above-mentioned competition for resources, the only way to reach a definite conclusion would be to present high distracting stimuli that activate differential neural populations.

Furthermore, our study suggests that selective attention to task relevant stimuli leads to a significant increase of the N170, which is in line with previous studies using similar paradigms [1,9]. However, other studies investigating the impact of selective attention on N170 amplitude in go/nogo, target detection, and repetition detection tasks with stimulus presentation durations ranging from 26 ms to 500 ms did not find such a modulation e.g. [32-35]. One possibility for these conflicting results might be the nature of the employed tasks: Our task required active encoding and subsequent maintenance of *every* task relevant stimulus and our behavioral data show high performance levels across subjects. In contrast, most of the other paradigms required simple identification of one or more predefined target stimuli. Unlike our study, the study using repetition detection [33] used simultaneous presentation of task relevant and task irrelevant stimuli to investigate the effect of selective attention on stimulus processing. In addition, task difficulty in this selective attention condition seems rather high (unfortunately, no behavioral data are provided) which might have influenced the collected data.

Another possible explanation for the increased N170 amplitudes to task relevant stimuli might be the need for stimulus discrimination when viewing these stimuli. Discriminating between stimuli has been shown to increase the posterior N1 [3] as compared to when participants were simply reacting to the onset of a stimulus. Since discriminative demands in our task were high, this might have influenced the obtained amplitudes. In addition, since task relevant and task irrelevant stimuli alternated in our paradigm, it was possible for participants to know in advance if the next stimulus would be relevant or irrelevant and distracting for successful task performance. This temporal expectation might have led participants to modulate their attention before the task irrelevant stimulus was actually presented. The enhanced amplitudes to task relevant stimuli might therefore represent the effect of a more general attention modulation induced by the structure of stimulus presentation instead of a specific effect of selective attention. This does not, however, explain the modulation observed for the task irrelevant stimuli. Amplitudes differed significantly depending on how distracting a task irrelevant stimulus was to successful task performance. Since the degree of distractibility of the task irrelevant stimuli varied randomly across trials, this modulation cannot be attributed to the structure of stimulus presentation.

In our study, we furthermore found correlations of ‘Hyperactivity/Restlessness’ and ‘Impulsivity/Emotional Lability’ scores with pooled N170 amplitudes irrespective of experimental condition, which are admittedly difficult to explain. One possibility is that participants with higher symptoms of hyperactivity and impulsivity might have been more easily bored by the task, which might be reflected in lower overall amplitudes. However, our task still appears to tap ADHD symptoms of ‘Inattention/Memory Problems’, ‘Hyperactivity/Restlessness’, and ‘Impulsivity/Emotional Lability’ that are unconnected to the investigated EEG components, as all symptom subscales were significantly correlated with task performance parameters.

Interestingly, a post-hoc analysis of P200 amplitudes after visual inspection of grand average waveforms showed a significant effect of stimulus relevance on mean amplitudes for this potential. Task relevant stimuli were associated with lower mean amplitudes while low distracting stimuli and passively viewed stimuli yielded higher mean amplitudes. There also appeared to be differential encoding of face and house stimuli as differences between distracting and passively viewed stimuli were found only for house stimuli. The finding of lower amplitudes for task relevant versus distracting stimuli is in line with the literature: Studies using visual search paradigms previously linked a posterior positivity between 200 ms and 300 ms to the suppression of distracting stimuli [36-38], while target stimuli were associated with a more negative potential [38]. In addition, a study using an auditory selective attention task reports increased P200 amplitudes to distracting stimuli after extended auditory attention training, suggesting an improvement of processing suppression for distractors [39]. However, all of these studies rely on simultaneous presentation of targets and distracting stimuli. Although our results also point to a stronger negativity for target stimuli and to an increased positivity for distracting stimuli, there are to our knowledge no studies with similar designs that these results could be critically compared to. Our results also show a differential effect for face versus house stimuli, which warrants further investigation beyond the scope of this study. Furthermore, studies using visual search paradigms often examined electrode positions PO7/PO8 [36,38,40], which were not part of our electrode layout. Although the electrodes analyzed in our study are close to these positions, the results for PO7/PO8 might differ from our results to an unknown extent

There are several limitations to this study: As stated above, it might be necessary to include high distracting stimuli from a separate category to disentangle possible explanations for any potentially present processing suppression. In addition, the passive viewing baseline condition might have been less arousing than the experimental conditions, which might have impacted on N170 amplitudes [41-44]. Although Vogel and Luck [3] did not find increased arousal to enhance posterior N1 amplitudes, future studies should ensure comparable arousal levels across the different conditions, possibly by including a target detection task in the passive viewing condition. In addition, since the sample consisted of healthy participants, no definite conclusions about clinical samples with regard to the reported correlations with ADHD symptomatology can be made. The task seems to tap ADHD symptomatology that is – contrary to our hypotheses – not reflected in the ERP data, possibly because overall ADHD symptomatology in the investigated sample was rather low. In the future, it would therefore be worthwhile to investigate participants meeting full diagnostic criteria for ADHD and possibly also looking for differences among the three diagnostic subtypes.

## Conclusions

In addition to partly replicating previous studies that showed early visual processing enhancement of task relevant and suppression of task irrelevant stimuli, we could extend and clarify these findings. Our results point to an enhancement of early visual processing of task relevant stimuli and to a possible suppression or absent enhancement of processing of task irrelevant stimuli – if these stimuli are high distracting to successful task completion. The efficiency of this processing modulation seems to have direct behavioral consequences. An extension of this research to clinical populations could yield results that might help improve models of clinical disorders as well as models of the concepts of cognitive control and selective attention.

## Methods

### Task

The experimental task consisted of a 1-back paradigm with alternately presented task relevant and task irrelevant stimuli [see 9 for a similar version of this task]. Our task employed pictures of neutral faces taken from the FERET database [45] and pictures of German houses without any prominent distinguishing features. For lack of an existing database, the house pictures were taken in a rural area in southeast Germany.

The experiment consisted of three conditions: Two experimental conditions (“houses relevant” and “faces relevant”) and a passive viewing baseline condition. Each condition was presented twice, yielding a total of six blocks containing eighty stimuli each. Of these eighty stimuli, forty (i.e. 50% of all stimuli presented in the block) were task relevant and forty (i.e. another 50%) were task irrelevant distractors. For the two experimental conditions, the forty relevant stimuli were all from the same category (i.e. 100% “face” or 100% “house” stimuli). In contrast, the forty task irrelevant stimuli were split evenly to be from the same category as the task relevant stimuli (yielding twenty high distracting stimuli, i.e. 50% of all task irrelevant stimuli were high distracting) or from another category (yielding twenty low distracting stimuli, i.e. 50% of all task irrelevant stimuli were low distracting). The passive viewing baseline condition always contained forty house (i.e. 50% of all presented stimuli) and forty face stimuli (i.e. another 50%), which – only in this condition – were presented in random order.

During each “houses relevant” and “faces relevant” condition, five task relevant stimuli (i.e. 12.5% of all task relevant stimuli presented in the block) were repeated in a 1-back fashion requiring a behavioral response. Three task relevant stimuli (i.e. 7.5% of all task relevant stimuli) were repeated in a 2-back fashion not requiring a behavioral response. The 2-back repetitions were included to ensure that participants would not simply react to the familiarity of a stimulus. All repeated stimuli were excluded from later EEG data analysis. On a behavioral level, reaction times for correct responses as well as number of false alarms and number of missed target stimuli were recorded. Every picture was shown only once in one of the three conditions. Task irrelevant stimuli never required a behavioral response.

Although task relevant and task irrelevant stimuli were presented alternately, the task relevance or task irrelevance of a stimulus was additionally denoted by two horizontal or vertical bars in each of the four corners of the display (see Figure 1 for an example of the “faces relevant” condition). Participants were instructed beforehand about the markings (e.g. horizontal bars marking task relevant stimuli and vertical bars marking task irrelevant stimuli – this was counterbalanced across participants) and markings were kept consistent across the entire experiment. All stimuli were presented for 1,000 ms with the interstimulus interval showing a grey fixation cross and ranging from 1,750 ms to 2,750 ms. This experimental set-up led to four different relevance-levels of the presented stimuli: task relevant stimuli, high distracting task irrelevant stimuli, low distracting task irrelevant stimuli, and passively viewed stimuli.

Participants were seated 50 cm from the monitor and viewed stimuli with approximately 10 cm height by 7.5 cm width. The whole display including the markings was 12 cm by 12 cm, subtending 14° of visual angle.

### Participants

Fifty participants took part in this study. They were recruited from a previously established subject pool see also [46] as well as through university advertisement. Participants were mostly students and received 12€ as compensation for their participation. All participants were right-handed, had normal or corrected-to-normal vision, and were free of neurological or psychiatric diseases. Due to a technical error during data collection, only forty percent of the original sample (twenty participants; mean age 25.4 ± 4.1 years) could be fully analyzed. Ethical approval was obtained through the Ethical Review Board of the medical faculty of the University of Würzburg; all procedures involved were in accordance with the 2008 Declaration of Helsinki. Participants gave written informed consent after full explanation of procedures.

### Psychological assessment

Participants completed three ADHD questionnaires to assess individual symptoms of both childhood and adult ADHD: The Adult ADHD Self-Report Scale (ASRS) [47] is an 18-item questionnaire assessing ADHD symptoms based on the DSM-IV-TR [48]. Participants were prescreened and selected based on their ASRS scores to ensure variability of ADHD symptoms in the sample. All participants had either a score of ten or lower on both the inattention and the hyperactivity scales or a score of at least 15 on any one of the two scales. The Conners’ Adult ADHD Rating Scales (CAARS) [49] is a more refined questionnaire, adding symptoms of adult ADHD to the core ADHD symptoms stated in the DSM-IV-TR [50,51]. To ensure that no participant met diagnostic criteria of childhood ADHD as described in the DSM-IV-TR [48], participants also completed the Wender Utah Rating Scale (WURS) [52]. No participant scored above the cut-off score for the short version [53] of this questionnaire. To control for affect and depressive symptoms, subjects furthermore completed the Positive and Negative Affect Schedule (PANAS) [54,55] and the Beck Depression Inventory (BDI-II) [56].

### Electrophysiological recording and data analysis

Event-related potentials (ERPs) were recorded from 28 Ag/AgCl active electrodes, which were placed according to the 10–20 guidelines using the actiCap system. Additional electrodes were placed under the right eye as well as on both outer canthi to monitor eye movement. The ground electrode was placed at AFz. Impedance was kept below 10 kΩ for all electrodes. Data was recorded with the software Brain Vision Recorder 1.20 (Brain Products GmbH; Munich, Germany) in relation to a midline reference electrode placed at FCz with a sampling rate of 1000 Hz.

The data was analyzed with the software Brain Vision Analyzer 1 (Brain Products GmbH). Band-pass filters were set to 0.1-30 Hz, with a 50 Hz notch filter. Eye movement artifacts were corrected [57] and the data was re-referenced to an average recorded reference. Stimulus-locked EEG epochs from -100 ms to 500 ms were segmented for the different stimuli. All stimuli used for 1- or 2-back repetitions as well as segments containing false alarm responses were excluded from further analysis. The data was baseline corrected to the mean amplitude from -100 ms to 0 ms. Epochs containing artifacts with the voltage in any channel exceeding ±100 μV or showing drops or rises of more than 100 μV/ms were rejected and artifact-free epochs were averaged.

Based on the literature [2,9,11,58] and grand average topography, channels O1 and O2 were selected for P100 analysis, and channels P7/P8, and PO9/PO10 were chosen for analysis of the N170. Based on the grand average time curve over all participants, the P100 was defined as the most positive peak in the time window from 70 ms to 140 ms. The N170 was defined as the most negative peak in the time window from 140 ms to 210 ms. Peaks were automatically detected and manually adjusted if necessary. In addition, a post-hoc analysis of the P200 defined as the mean amplitude in the time window from 210 ms to 270 ms in the channels P7/P8 and PO9/PO10 was carried out. Peak and mean amplitudes, respectively, were then exported for subsequent analysis with SPSS Statistics 20 (IBM®, New York, USA).

### Statistical analysis

ERP amplitudes were analyzed separately for the P100 and the N170 by using a repeated measures analysis of variance (ANOVA). The ANOVA for the P100 comprised the within-subject factors hemisphere (left, right), stimulus category (face, house), and task relevance (relevant, irrelevant – high distracting, irrelevant – low distracting, passively viewed). The ANOVA for the N170 included the within-subject factors hemisphere (left, right), channel group (P7/P8, PO9/PO10), stimulus category (face, house), and task relevance (task relevant, task irrelevant – high distracting, task irrelevant – low distracting, passively viewed). Hypotheses-driven one-sided t-tests were used for the factors task relevance and stimulus category; two-tailed t-tests were used for all other post-hoc comparisons. To control for multiple comparisons, all post-hoc t-tests were Šidák-corrected. Degrees of freedom were adjusted according to Greenhouse-Geisser, if assumptions of sphericity were not met. Effect sizes were calculated based on average means and standard deviations and corrected for correlation among the means using G*Power 3.0.3 [59].

In addition, the following exploratory correlation coefficients were calculated: The N170 component pooled across the four task conditions (task relevant, task irrelevant – high distracting, task irrelevant – low distracting, passively viewed) was correlated with the three CAARS subscales incorporating symptoms of adult ADHD (‘Inattention/Memory Problems’, ‘Hyperactivity/Restlessness’, ‘Impulsivity/Emotional Lability’), yielding three correlation coefficients. The N170 difference amplitude of task relevant minus high distracting task irrelevant stimuli was correlated with the same three CAARS subscales, yielding again three correlation coefficients. In addition, the N170 difference amplitude of task relevant minus high distracting task irrelevant stimuli was correlated with the behavioral measures ‘percentage of missed targets’ and ‘number of false alarms’, yielding two correlation coefficients. These behavioral measures were also correlated with the three above-mentioned CAARS subscales, yielding another six correlation coefficients. This procedure resulted in a total of fourteen correlations coefficients that were tested for significance.

The ANOVA for the P200 was identical to the ANOVA used for the N170 with the within-subject factors hemisphere (left, right), channel group (P7/P8, PO9/PO10), stimulus category (face, house), and task relevance (task relevant, task irrelevant – high distracting, task irrelevant – low distracting, passively viewed). Since this ANOVA was conducted post-hoc, all post-hoc t-tests were two-tailed and Bonferroni-corrected.

For all analyses, *p*-values below .05 were considered significant.

## Competing interests

The authors declare no competing interests.

## Authors’ contributions

SCB designed the study, participated in data analysis and interpretation, and drafted the manuscript. A-CE participated in data analysis and revised the manuscript. LDM supervised data collection and revised the manuscript. AN participated in data collection and data analysis. PP participated in the design of the study and data interpretation. MJH participated in the design of the study and data analysis. All authors read and approved the final manuscript.
